# Quantitative Colocalization Analysis in Fluorescence Microscopy

**DOI:** 10.21769/BioProtoc.5832

**Published:** 2026-10-20

**Authors:** Charles James Myers, Beiyan Nan

**Affiliations:** Department of Biology, Texas A&M University, College Station, TX, USA

**Keywords:** Colocalization, Fluorescence microscopy, Quantification, Pearson’s correlation, Manders’ correlation

## Abstract

Spatial organization of macromolecules is fundamental to cellular function, with colocalization providing key insights into molecular interactions and biological processes. However, quantification remains challenging due to diverse localization patterns and irregular sample geometries. Here, we present a protocol for analyzing colocalization between two fluorescent probes using coAnalyzer, a MATLAB-based software package. coAnalyzer features a user-friendly graphical interface and supports region of interest (ROI) selection, image merging, line scanning, signal isolation, scatterplot generation, and quantitative analysis. coAnalyzer enables colocalization analysis of any two fluorophores, regardless of the proteins or dyes involved. Its broad applicability across a wide range of organisms and sample types demonstrates the robustness, flexibility, and versatility of the platform.

Key features

• Provides both quantitative and semi-quantitative colocalization analysis using multiple coefficients.

• Features a user-friendly graphical user interface (GUI) that provides step-by-step visualization of the analysis process.

• Supports 1D (line scan) and 2D analysis.

• Analyzes large datasets and generates histograms for effective data presentation.

## Graphical overview


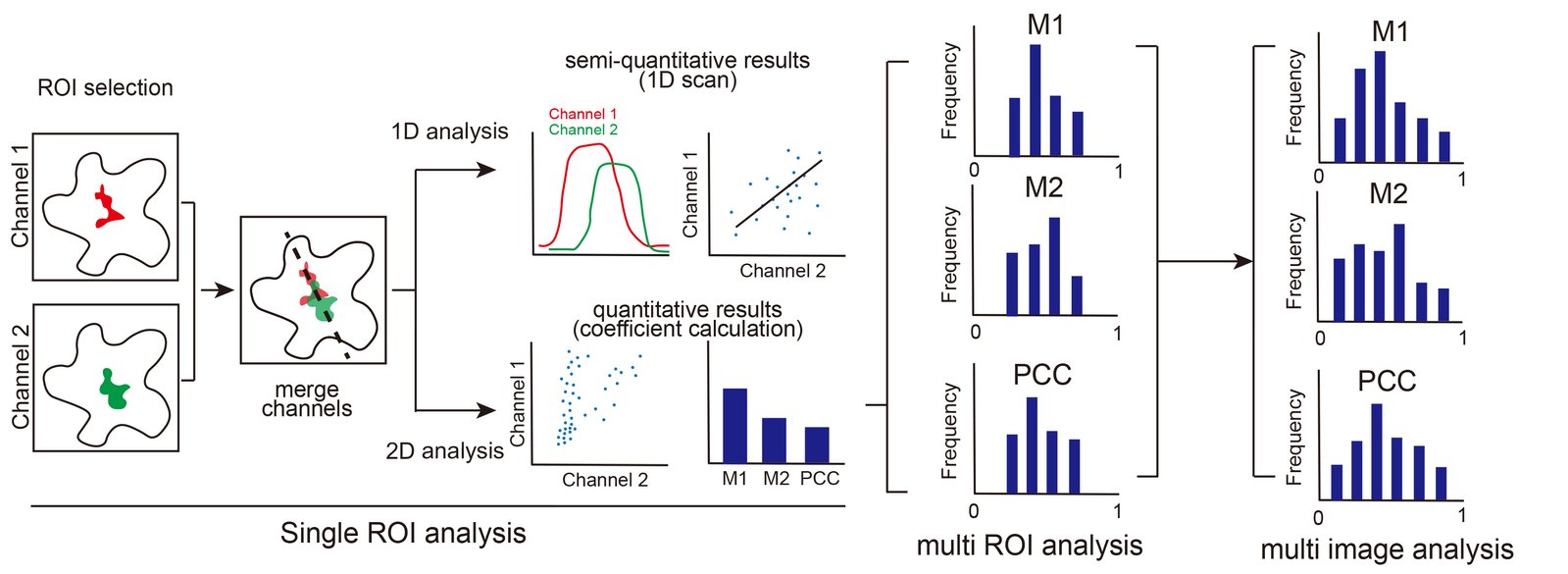


**Workflow of coAnalyzer, a MATLAB-based software package that analyzes colocalization in single regions of interest (ROIs), multiple ROIs, and multiple images.** Images are for schematic presentation only.

## Background

The intricate spatial organization of macromolecules, such as proteins and nucleic acids, plays a central role in living systems. Macromolecules are organized through various mechanisms at different levels. First, for multicellular organisms, the production of many molecules is usually cell- or tissue-specific. Second, functional molecules often localize to specific organelles or subcellular locations in individual cells. Third, multiple molecules also form complexes through physical or biochemical interactions. Thus, decoding the localization of macromolecules, especially their colocalization with cellular structures and other macromolecules, provides critical information on their biological functions. Fluorescence microscopy has become an invaluable tool in cell biology because of its label specificity, sensitivity, and non-invasive nature. Using fluorescence microscopy, different molecular or cellular components can be labeled with distinct fluorophores, their localization imaged in separate channels, and their colocalization quantified by the overlap of their fluorescence signals [1].

Colocalization consists of two distinct components: co-occurrence and correlation, which reflect the extent of spatial and intensity co-distribution of two fluorophores, respectively [2,3]. To display colocalization qualitatively, the prevalent method is to show the superposition of individual fluorescent images obtained in different channels and to reflect the overlap of different fluorophores using the colors of the merged image. Scatterplots provide a semi-quantitative method for visualizing colocalization, in which the intensities of two fluorophores are plotted against each other pixel-by-pixel. Under proportional co-distribution, the points in a scatterplot would cluster around a straight line. In contrast, points would show a spread distribution in the case of a lack of co-distribution [2–4]. However, superposition and scatterplot do not provide quantitative measurements and cannot be used for comparing the extent of colocalization across different genetic backgrounds and experimental conditions.

**Manders’ colocalization coefficients (M1 and M2)**

M1, the fraction of color R overlapping with color G

$$M1=\frac{\sum R_{i,\;colocal}}{\sum R_{i}}$$ (1)

Where R_i_, colocal = R_i_, if G_i_ > 0, and R_i_, colocal = 0 if G_i_ = 0.

M2, the fraction of color G overlapping with color R

$$M2=\frac{\sum G_{i,\;colocal}}{\sum G_{i}}$$ (2)

Where G_i_, colocal = G_i_, if R_i_ > 0, and G_i_, colocal = 0 if R_i_ = 0.

**Pearson’s correlation coefficient (PCC)**

$$PCC=\frac{\sum (R_{i}-\overset{-}{R})\times (G_{i}-\overset{-}{G})}{\sqrt{\sum {{(R}_{i}-\overset{-}{R})}^{2}\times \sum ({G_{i}-\overset{-}{G})}^{2}}}$$ (3)

Where R_i _and G_i_ represent the intensity values of two individual channels of pixel i, and $\overset{-}{R}$ and $\overset{-}{G}$ represent the mean intensities of these channels across the entire image.

**Manders’ overlap coefficient (MOC)**

$$MOC=\frac{\sum (R_{i}\times G_{i})}{\sqrt{\sum {R_{i}}^{2}\times \sum {G_{i}}^{2}}}$$ (4)

To quantify the co-occurrence, M1 and M2 are the most popular metrics. M1 and M2 measure the co-occurrence fraction of color 1 with color 2 (M1) and the co-occurrence fraction of color 2 with color 1 (M2) [5]. The calculation of M1 and M2 requires the sum of the intensity for each fluorophore and the sum of the intensity for each fluorophore within the area where these two fluorophores overlap [5]. Thus, M1 and M2 provide intuitive and direct measurements of colocalization, which are independent of signal proportionality.

Pearson correlation coefficient (PCC) and Mander’s overlap coefficient (MOC) are widely used in the quantification of correlation. PCC quantifies to what extent the signal intensity variation in two color images can be explained by a simple, linear relationship [3]. PCC measures the pixel-by-pixel covariance between the two-color images, which is sensitive to both signal co-occurrence and correlation. The meaning of extremely high or low PCC is generally clear, but intermediate values are difficult to interpret. Values close to 1 or -1 indicate that the two-color images have a perfect positive or negative correlation, and values close to 0 indicate a lack of correlation [3]. Mander’s overlap coefficient (MOC) is mathematically similar to PCC. Regarding the analysis of colocalization from fluorescence images, the major difference between these two metrics is that MOC uses absolute fluorescence intensities, while PCC is calculated from the intensities after the subtraction of the mean [2]. Compared to PCC, MOC is almost independent of signal proportionality and sometimes measures co-occurrence indirectly and unpredictably [2,3]. Therefore, we choose co-occurrence fractions (M1 and M2) and PCC as the major indicators for colocalization.

Various software packages perform colocalization analysis using these metrics. However, many of these tools operate as a “black box,” providing little or no visualization of intermediate processing steps. As a result, users often determine signal thresholds through trial and error, leading to subjective, arbitrary, and potentially irreproducible results. Here, we present a protocol for analyzing colocalization in fluorescence microscopy images using coAnalyzer, a MATLAB-based software package. The graphical user interface (GUI) enables multiple ROI selection, one-step image merging, line scanning, data integration, and stepwise visualization of the analysis process. Additionally, coAnalyzer can be applied to image folders, facilitating efficient analysis of large datasets.

## Software and datasets

1. Developed MATLAB-based script for colocalization analysis (https://github.com/NanLabMyxo/coAnalyzer), DOI: 10.5281/zenodo.21516437

2. MATLAB R2018b and later versions (The MathWorks, Inc. https://www.mathworks.com/help/install/ug/install-products-with-internet-connection.html) or MATLAB Online (https://www.mathworks.com/products/matlab-online.html)

3. Operating systems: Microsoft Windows 11 Enterprise version 10.0 (Build 26200, 26100), Microsoft Windows 10 Enterprise version 10.0 (Build 19045)

3. Image Processing Toolbox (https://www.mathworks.com/products/image-processing.html, version 26.1)

4. Optimization Toolbox (https://www.mathworks.com/products/optimization.html, version 26.1)

5. Statistics and Machine Learning Toolbox (https://www.mathworks.com/products/statistics.html, version 26.1)

*Note: The MATLAB toolboxes indicated are free and available on the MATLAB Online platform. Users must use compatible MATLAB toolboxes for the MATLAB program (e.g., MATLAB toolbox version 26.1 is compatible with MATLAB R2026a).*

## Procedure

**A. Load and label images**

1. Start MATLAB and load the “co3.fig” file. The interface of the GUI of coAnalyzer will be displayed (Figure 1).

*Note: Users should add the downloaded coAnalyzer folder to the MATLAB path and run the “co3.fig” file to launch the GUI of coAnalyzer.*

**Critical:** The Image Processing Toolbox and the Optimization Toolbox must be installed before launching the software package.

**Figure 1.**
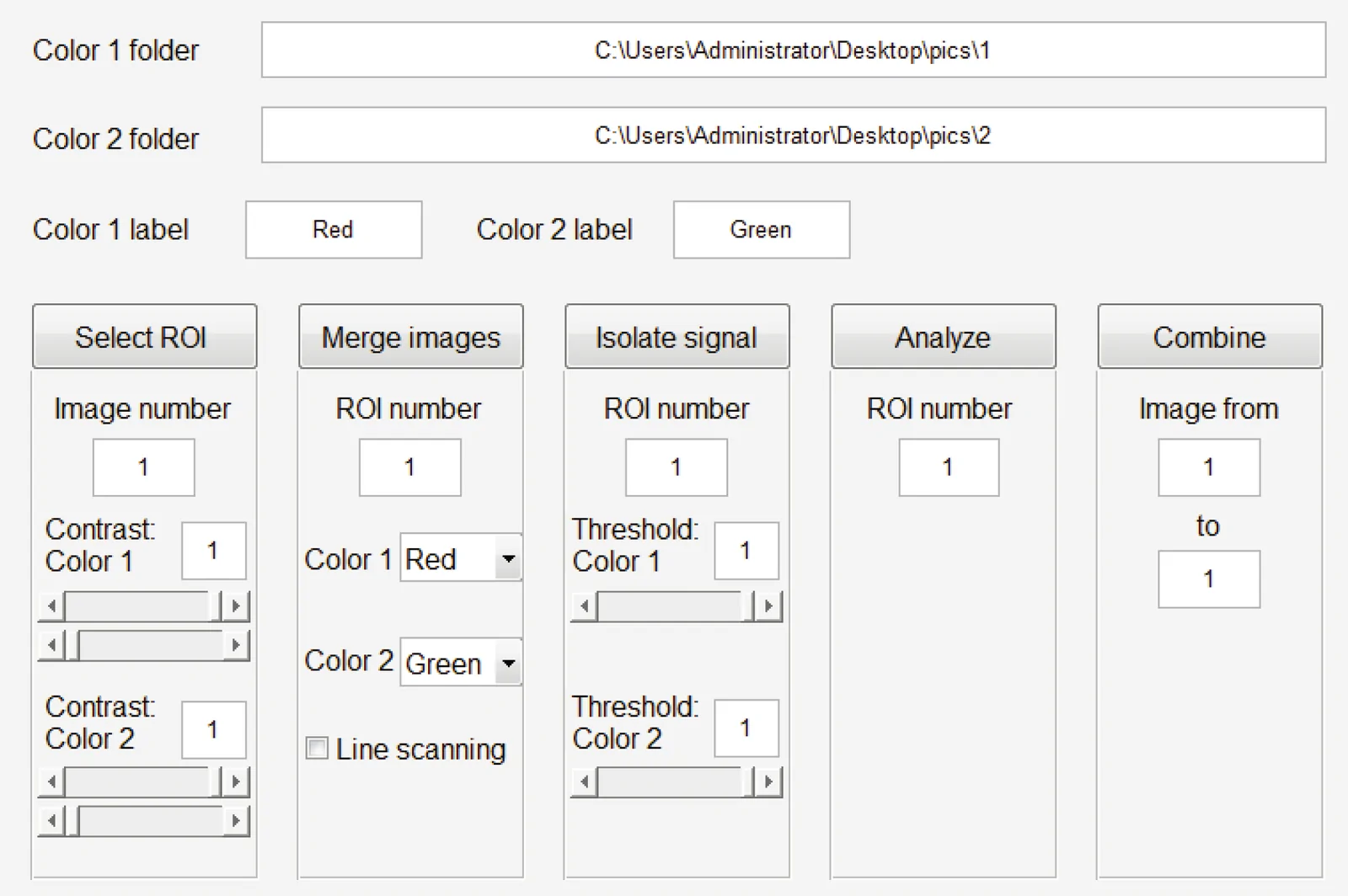
GUI of coAnalyzer

2. In the *Color 1 folder* and *Color 2 folder* text boxes, type or copy and paste the folder paths for images from the first and second imaging channels.

*Notes:*

*1. A list of input file formats supported and not supported by the software package is included in Table 1.*

*2. A list of output files, their locations, descriptions, and file formats after image analysis is included in Table 2.*

*3. Image files in both folders should contain matched and identically named/ordered images. The program automatically pairs images across both channels.*

*4. Images used in the procedure section were acquired using emission wavelengths corresponding to the respective fluorophores and fluorescent dyes. Single image files of 8- or 16-bit depth are supported by the software package.*

**Table 1.** Input file formats supported and not supported by coAnalyzer. Input file formats with their extensions and software compatibility.

File format	Extension(s)	Software compatibility
Tagged Image File Format (TIFF)	.*tif, .tiff*	Supported
Joint Photographic Experts Group (JPEG)	.*jpg, .jpeg*	Supported
Portable Network Graphics (PNG)	.*png*	Supported
Bitmap Image File (BMP)	.*bmp*	Supported
Graphics Interchange Format (GIF)	.*gif*	Supported
Portable Graymap File Format (PGM)	.*pgm*	Supported
Flexible Image Transport System (FITS)	.*fits*	Supported
Analyze 7.5	.*img, .hdr*	Not supported
Nikon NIS-Elements Dimensions Images	.*nd2*	Not supported
Microsoft Paint Project File	.*paint*	Not supported

*Note: Hyperstack files are not compatible with coAnalyzer.*

**Table 2.** Output files generated by coAnalyzer during image analysis. The table lists each output file with its storage location, description, and file format.

Analysis step	Output file(s)	Output location	Description	File extensions
ROI selection	ROIplot	*Color 1 folder* → image-specific data directory	Image showing the selected ROIs for analysis	.*tif*
	ROIplot2	*Color 1 folder* → image-specific data directory	Provides an additional ROI selection visualization	.*tif*
	mask	*Color 1 folder* → image-specific data directory	Binary mask defining the selected ROI	.*tif*
	mask1, mask2, …	*Color 1 folder* → image-specific data directory	Individual ROI masks and associated mask data for each ROI	.*tif, .txt*
Image merging	Merged1, Merged2, …	*Color 1 folder* → image-specific data directory	Merged two-channel fluorescence images of each ROI for visual assessment of colocalization	.*tif*
Line scanning	LineScan1, LineScan2, …	*Color 1 folder* → image-specific data directory	Line-scan profile images generated from the line-scan path	.*tif*
	LineTwoColor1, LineTwoColor2, …	*Color 1 folder* → image-specific data directory	Two-channel line-scan images showing signal distribution across both fluorescence channels	.*tif*
	MergedWithLine1, MergedWithLine2, …	*Color 1 folder* → image-specific data directory	Merged two-channel fluorescence images with the line-scan path	.*tif*
	(Color 1 label) LineInt1, (Color 1 label) LineInt2, …	*Color 1 folder* → image-specific data directory	Line-scan intensity values extracted from the Color 1 channel	.*txt*
	(Color 2 label) LineInt1, (Color 2 label) LineInt2, …	*Color 1 folder* → image-specific data directory	Line-scan intensity values extracted from the Color 2 channel	.*txt*
Signal isolation	bw(Color 1 label)1, bw(Color 1 label)2, …	*Color 1 folder* → image-specific data directory	Thresholded binary images isolating the signal from the Color 1 channel	.*tif*
	bw(Color 2 label)1, bw(Color 2 label)2, …	*Color 1 folder* → image-specific data directory	Thresholded binary images isolating the signal from the Color 2 channel	.*tif*
	Thresholdingplot1, Thresholdingplot2, …	*Color 1 folder* → image-specific data directory	Images showing the thresholding results used for signal isolation	.*tif*
Colocalization analysis	Scatterplot1, Scatterplot2, …	*Color 1 folder* → image-specific data directory	Pixel-intensity scatterplots comparing the two fluorescence channels	.*tif, .eps*
	barplot1, barplot2, …	*Color 1 folder* → image-specific data directory	Bar plots summarizing M1, M2, and PCC measurements for each ROI	.*tif, .eps*
	M1 (Color 1 label)1, M1 (Color 1 label)2, …	*Color 1 folder* → image-specific data directory	M1 values for each ROI in the Color 1 channel	.*txt*
	M2 (Color 2 label)1, M2 (Color 2 label)2, …	*Color 1 folder* → image-specific data directory	M2 values for each ROI in the Color 2 channel	.*txt*
	PCC1, PCC2, …	*Color 1 folder* → image-specific data directory	PCC values for each ROI	.*txt*
	(Color 1 label) Int1, (Color 1 label) Int2, …	*Color 1 folder* → image-specific data directory	Pixel-intensity values extracted from the Color 1 channel	.*txt*
	(Color 2 label) Int1, (Color 2 label) Int2, …	*Color 1 folder* → image-specific data directory	Pixel-intensity values extracted from the Color 2 channel	.*txt*
Combining output	hist	*Color 1 folder* → sum directory	Summary histogram of M1, M2, and PCC measurements across analyzed images	.*tif, .fig*
	M1	*Color 1 folder* → sum directory	Combined M1 values across analyzed images/ROIs	.*txt*
	M2	*Color 1 folder* → sum directory	Combined M2 values across analyzed images/ROIs	.*txt*
	PCC	*Color 1 folder* → sum directory	Combined PCC values across analyzed images/ROIs	.*txt*

*Note: Color 1 label and Color 2 label refer to the user-defined names assigned to the two fluorescence channels. Numbered output files correspond to individual ROIs analyzed by coAnalyzer.*

3. In the *Color 1 label* and *Color 2 label* text boxes, type the color labels of the respective imaging channels. These labels will be displayed in the analysis result windows and used as identifiers for M1 and M2.

*Note: Labels of image channels will be displayed in the result windows and in the names of a few output files (Table 2).*

4. In the *Image Number* text box below the *Select ROI* button, type “1” to begin the image analysis for the first image.

*Notes:*

*1. Once the user follows the steps below and finishes analyzing the first image, type “2” in the Image Number text box to analyze the second image. Repeat these steps to analyze all images in the folder. To allow users to control the analysis step-by-step, coAnalyzer requires manual image indexing.*

*2. Users must track which image number they are on. In the case where a non-existent image number is typed, an error message will be displayed: “error, image ‘x’ does not exist.”*

5. Adjust the contrast for images for Color 1 and Color 2 by dragging the scroll thumbs.

*Note: This step allows the user to adjust the contrast of both channels. The contrast adjustment is for visualization only and does not affect the quantitative results (M1/M2/PCC).*

**B. Select ROIs**

6. Click the *Select ROI* button. A ROI selection window (Figure 2) will appear. To select each ROI, draw a polygon to enclose it: click once to mark the first vertex of the polygon, and a dot will appear. Drag the cursor to the second vertex and click again. Repeat this process to close the polygon, double-click to confirm the selection of a ROI, and proceed to the next ROI (Figure 2). Each selected ROI will be marked with red lines and a red number. After selecting all the ROIs, press *Enter* to proceed to the next image number or step 7.

**Critical:** The Image Processing Toolbox and the Optimization Toolbox must be installed in MATLAB to perform this step.

*Notes:*

*1. ROIs can be selected in either imaging channel. Once selected, the program automatically reproduces the same selection in the other channel.*

*2. There are no universal standards on the minimum number of images and ROIs to be analyzed. Users should use statistical methods to justify their sampling choices.*

*3. Users can terminate ROI selection by pressing “F”.*

**Figure 2.**
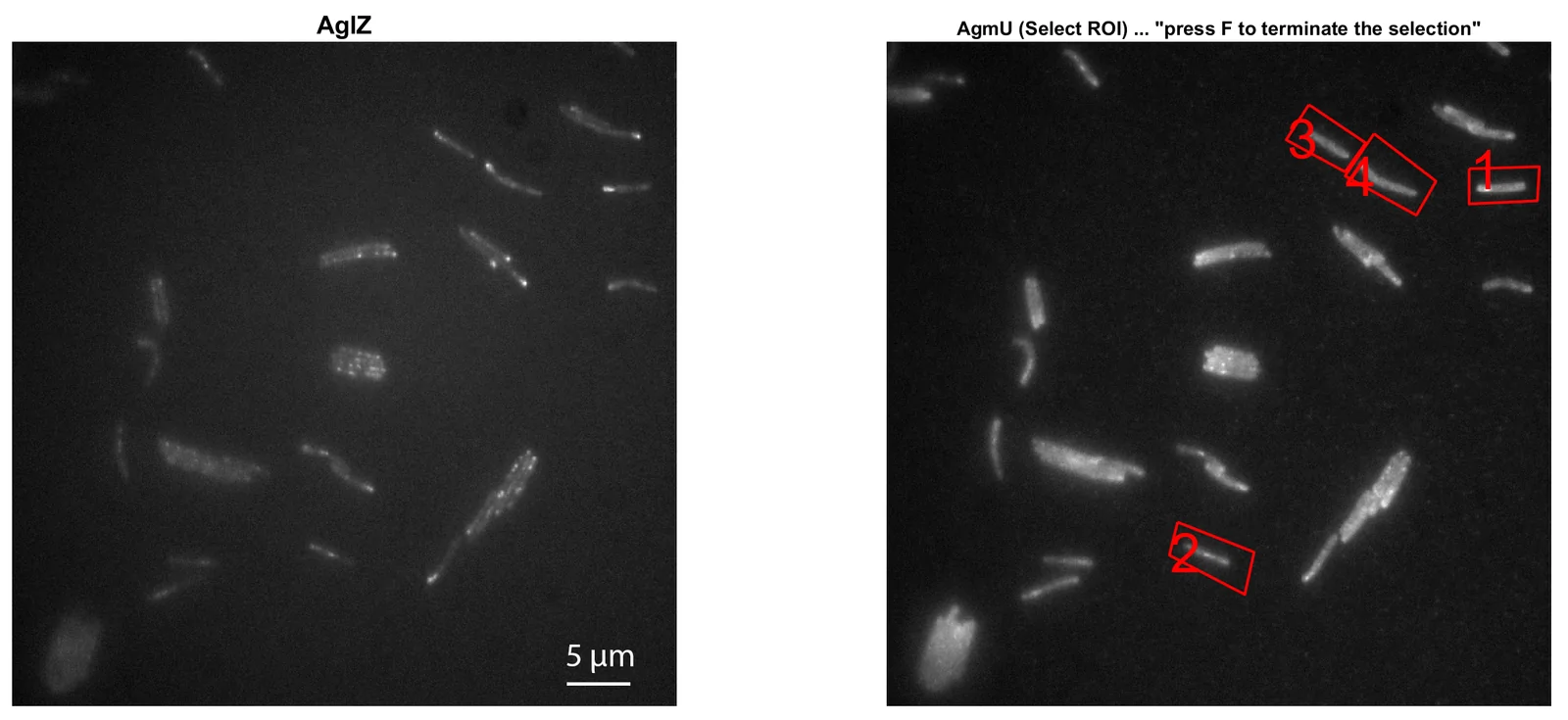
ROI selection. After clicking the *Select ROI* button, this window appears. Draw polygons to select ROIs on one image. Here, we used the model bacterium *Myxococcus xanthus* [6] that expresses two fluorescence-labeled motility proteins, AglZ-GFP and AgmU-mCherry. While AglZ is a cytoplasmic protein, AgmU localizes to both the cytoplasm and periplasm [7–9].

**C. Merge images**

7. The GUI of coAnalyzer (Figure 1) will reappear. Type “1” in the *ROI Number* text box under the *Merge images* button to begin the image analysis for the first ROI.

*Notes:*

*1. This number defines which ROI to be merged for analysis. There is no maximum number of ROIs that can be analyzed by the program.*

*2. Users should type the same ROI number consistently in ROI Number text boxes across* Merge images, Isolate signal, *and* Analyze *to merge images and perform 1D and 2D analysis for a specific ROI number.*

8. Under the *Merge images* button, select the colors of the first and second imaging channels from the drop-down menus.

*Note: These colors should correspond to the emission wavelengths used during imaging.*

9. Click the *Merge images* button to merge the images from the two imaging channels. A window (Figure 3A, D, G, J) will appear, which allows the user to assess the quality of the merged ROI. Close this window to continue.

**Critical:** To use the *line scanning* function (step 11), check the *Line scanning* box before clicking on the *Merge images* button.

10. The GUI of coAnalyzer (Figure 1) will reappear. Type “2” in the *ROI Number* text box under the *Merge images* button to merge the second ROI. Repeat steps 7–9 to merge multiple ROIs.

**Figure 3.**
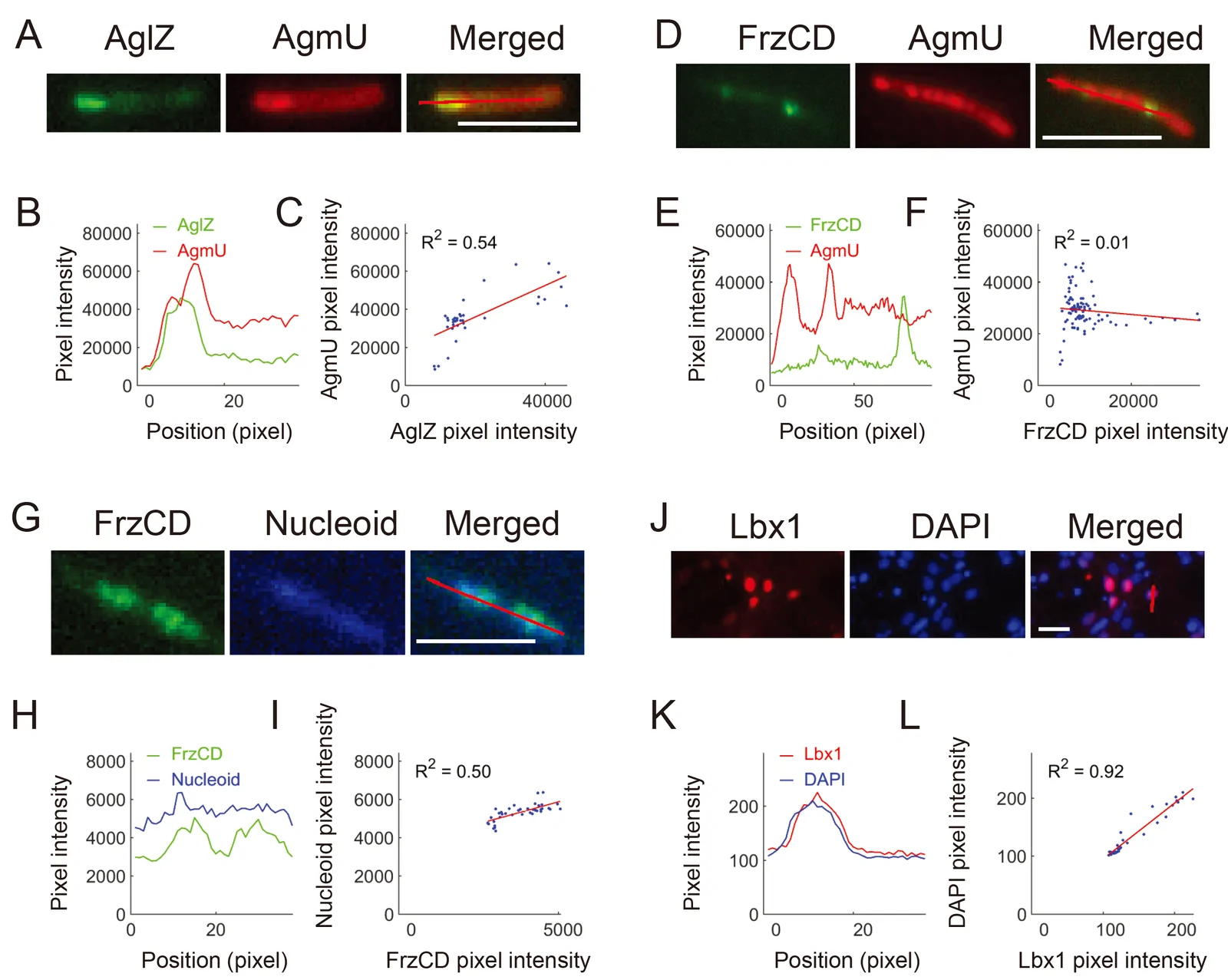
1D analysis. **(A, D, G, J)** After clicking the *Merge images* button, images from two channels will be merged. Each channel is displayed in the color defined by the user. A merged image allows the user to assess image quality. If the *Line scanning* box is checked before clicking the *Merge images* button, the user can define a line along which the fluorescence signals will be scanned. (**B–F, H–L**) Scanning results along individual lines. Panels **B, E, H, and K** show the fluorescence profile of each fluorophore, while panels **C, F, I, and L** display the correlation between the fluorescence intensities of each imaging channel. While FrzCD, a cytoplasmic chemoreceptor of *M. xanthus*, is labeled by GFP, chromosomal DNA (nucleoid) is stained by Hoechst. Lbx1 is a transcription factor marker found in the nucleus of expressing neurons in the mouse brain. A mouse brain tissue was used for the colocalization analysis between Lbx1-mCherry and DAPI-stained neurons. Scale bars, 5 μm.

**D. 1D analysis**

11. If the *Line scanning* function is selected, draw a straight line on the merged image (Figure 3A, D, G, J) across the region the user wishes to analyze, and double-click to confirm. A red line will appear on the merged image (Figure 3A, D, G, J). The line scanning results will be shown as the fluorescence profiles of the fluorophore from each fluorophore (Figure 3B, E, H, K) and their correlation along the line (Figure 3C, F, I, L). The results of each ROI are saved as files named “LineScan1, LineScan2, …” and “LineTwoColor1, LineTwoColor2, …” in the *Color 1 folder* under the data directory assigned for the specific image number. The LineScan file shows a plot of pixel intensities of the two fluorophores against their positions (pixel), and the LineTwoColor file shows a plot of the correlation between pixel intensity of one fluorophore against the other fluorophore. These files are saved in the TIFF file format.

*Notes:*

*1. The program automatically creates the data directories assigned for the image numbers in the Color 1 folder.*

*2.* Line scanning *is a tool for analyzing protein colocalization in one dimension. For analysis in two dimensions, skip this step and go to step 12.*

**E. 2D analysis**

12. Close the line scanning results window, and the GUI of coAnalyzer (Figure 1) will reappear. Type “1” in the *ROI Number* text box under the *Isolate signal* button to isolate the fluorescence signals for the first ROI.

*Note: This number defines which ROI will be analyzed.*

13. Adjust the threshold to isolate the fluorescence signals from images for Color 1 and Color 2 by dragging the scroll thumbs.

**Critical:** These adjustment buttons set a threshold to isolate fluorescence signals from images for Color 1 and Color 2. The threshold value is a normalized value from 0 to 1. For the best result, set the threshold at a value that best isolates the fluorescence signals from images without highlighting the image background as a signal. For consistency, use the same threshold value for all ROIs/images. The threshold value is displayed numerically in the text boxes above the scroll thumbs and can be applied across all ROIs, provided it is not changed manually by dragging the scroll thumbs.

14. Click the *Isolate signal* button. A result window (Figure 4A, D, G, J) will appear. Evaluate the fluorescence signals that were isolated from the current ROI. Close the signal isolation result window. The GUI of coAnalyzer (Figure 1) will reappear. Type “2” in the *ROI Number* text box under the *Isolate signal* button to continue the analysis for the second ROI. Repeat steps 13 and 14 to isolate the fluorescence signals from multiple ROIs.

**Pause point:** The user cannot proceed to *Analyze* (step 16) without isolating the fluorescence signals. When this step is skipped, the program warns the user by displaying the error message “error, ROI number ‘x’ does not exist.”

15. After signal isolation, the GUI of coAnalyzer (Figure 1) will reappear. Type “1” in the *ROI Number* text box under the *Analyze* button to analyze the first ROI.

*Note: This number defines which ROI to be analyzed.*

16. Click the *Analyze* button. 2D scatterplots (Figure 4B, E, H, K) and colocalization coefficients (Figure 4C, F, I, L) will appear. The results of each ROI are saved as files named “Scatterplot1, Scatterplot2, …” and “barplot1, barplot2, …” in the *Color 1 folder* under the data directory assigned for the specific image number. The “Scatterplot” file shows a scatterplot of the pixel intensity of one fluorophore against the other fluorophore, while the “barplot” file contains M1, M2, and PCC measurements of the two fluorescence signals. These files are saved in TIFF and Encapsulated PostScript file formats.

*Note: The program automatically creates the data directories assigned for the image numbers in the Color 1 folder.*

17. Close the results window. The GUI of coAnalyzer (Figure 1) will reappear. Type “2” in the *ROI Number* text box under the *Analyze* button to continue the analysis for the second ROI. Repeat steps 16 and 17 to analyze multiple ROIs.

*Note: The quantitative results of each ROI will be displayed separately. To combine the results from multiple ROIs, go to step 18.*

**Figure 4.**
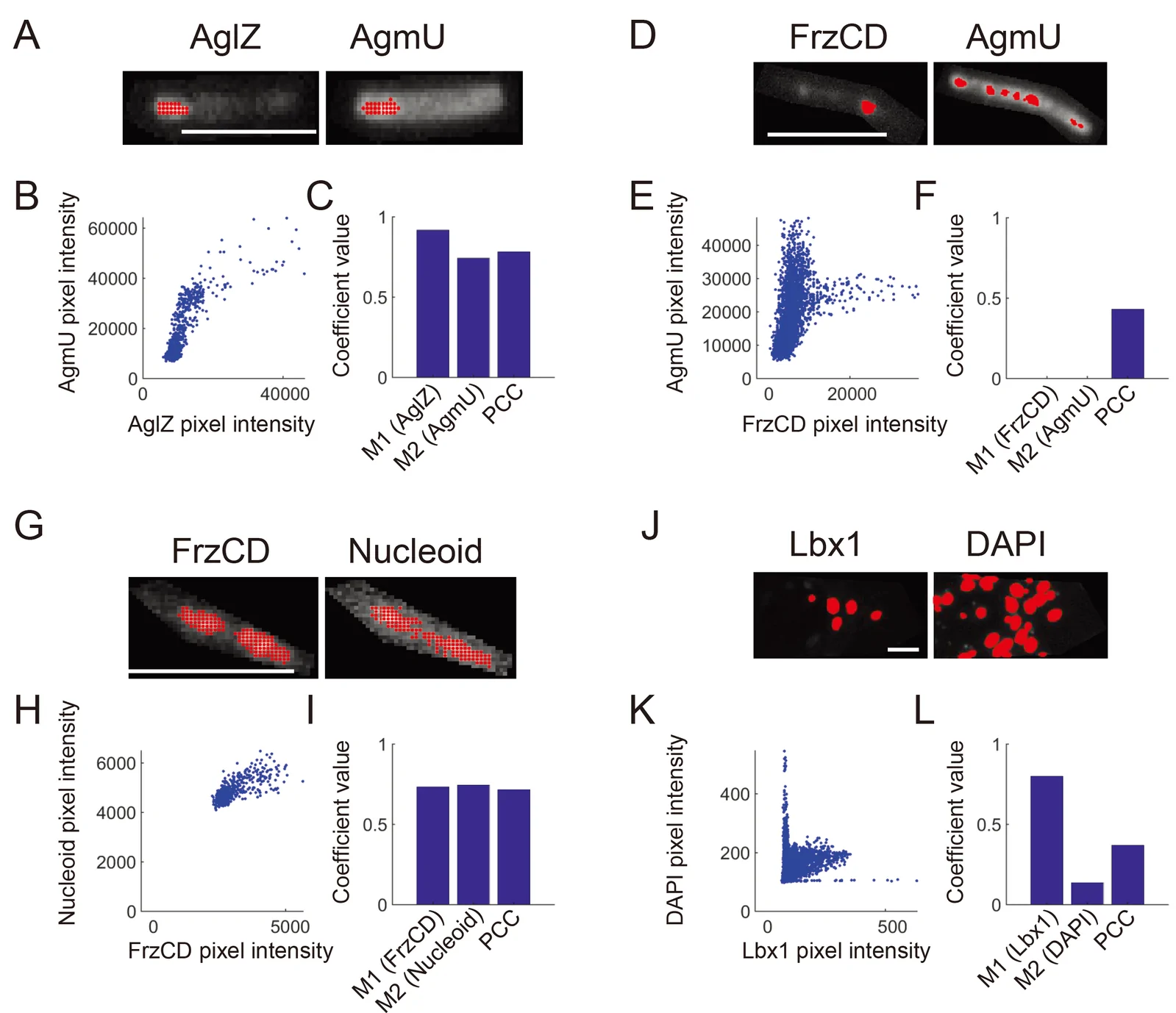
2D analysis of single ROIs. (A, D, G, J) Signal isolation results. **(B, E, H, K)** scatterplots of the pixel intensities of one fluorophore against the other. **(C, F, I, L)** Bar plots presenting the M1, M2, and PCC measurements of the two fluorescence signals. For additional details, see the Validation of protocol section. Scale bars, 5 μm.

**F. Combine results from multiple ROIs/images**

18. After analyzing all the ROIs, the GUI of coAnalyzer (Figure 1) will reappear. Type “1” in both the *Image from* and *image to* text boxes under the *Combine* button to combine the results from all ROIs within image number 1. To combine the results from multiple images, type the numbers of the first and last images into the *Image from* and *image to* text boxes, respectively.

*Note: For instance, to combine all the ROIs in images 1 and 2, type “1” and “2” into the* Image from *and* image to *text boxes.*

**Pause point:** All images must be fully analyzed before combining results. If unanalyzed images are combined, the program will display the error message “error, data for image ‘x’ does/do not exist”.

19. Click the *Combine* button. The result window for combined results from multiple ROIs/images (Figure 5) will appear. This graphical result is automatically saved as a file named “hist.tif.” under the “sum” directory in the *Color 1 folder*. Numerical data are saved in files named “M1.txt.” and “M2.txt.” for Manders’ colocalization coefficients and “PCC.txt.” for Pearson’s correlation coefficient (PCC). These files contain the colocalization coefficient values calculated for each ROI in all the analyzed images in the *Color 1 folder* (one value per ROI). Colocalization coefficients for individual ROIs in each image can be found under the data directory assigned for the specific image number in the *Color 1 folder*. These files are saved in the .txt file format.

*Note: The program automatically creates the* sum *directory and the data directories assigned for the image numbers in the Color 1 folder.*

**Figure 5.**
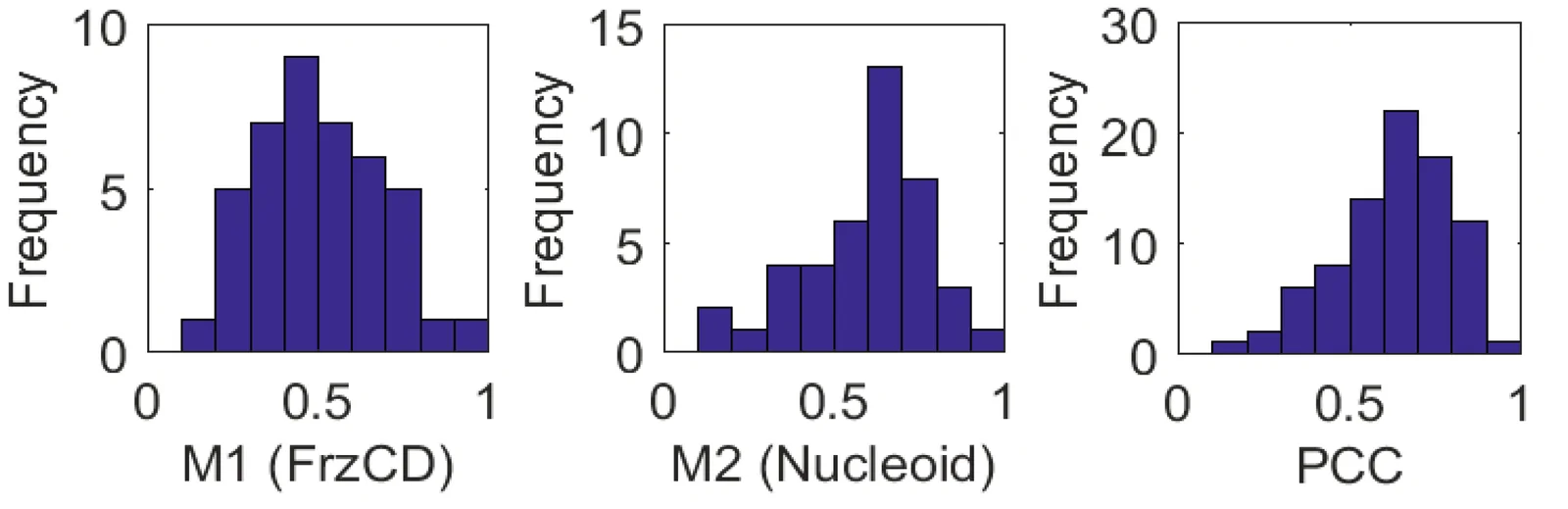
Results for combining multiple ROIs/images. Here, we show the colocalization of GFP-labeled FrzCD and Hoechst-stained DNA (nucleoid). Histograms of M1, M2, and PCC are calculated from 42 cells in 20 images.

## Validation of protocol

We used the colocalization between the proteins AglZ and AgmU (*alias* GltD) in the rod-shaped gliding bacterium *Myxococcus xanthus* to validate coAnalyzer [9]. Previous reports showed that fluorescence-labeled AglZ and AgmU both assembled in the gliding motor complexes, which formed clusters along the cell body [7]. We imaged *M. xanthus* cells that express AglZ-GFP and AgmU-mCherry and visualized their colocalization using coAnalyzer. Merged images show that the localization patterns of the GFP-labeled AglZ and mCherry-labeled AgmU largely overlap in *M. xanthus* cells (Figure 3A and Figure 5 in [7]), which is confirmed by the fluorescence profiles generated by the *line scan* function (Figure 3B and Figure 5 in [7]). Using the same function, the correlation between the fluorescence intensities of AglZ-GFP and AgmU-mCherry along the user-defined line was further calculated, where R^2^ > 0.5 indicates significant correlation between these two proteins but nonidentical localization (Figure 3C and Figure 5 in [7]). In contrast, AgmU and FrzCD, a cytoplasmic chemoreceptor, occupy mutually exclusive positions in *M. xanthus* cells [7]. Merged images and line scanning results indicate that the localization patterns of FrzCD-GFP and AgmU-mCherry do not overlap (Figure 3D–F and Figure 5 in [7]). A study by Moine et al. [10] reported that FrzCD binds the nucleoid of *M. xanthus* with its N-terminal domain. We imaged FrzCD-GFP and Hoechst-stained nucleoid in *M. xanthus* cells and tested coAnalyzer for its ability to analyze the colocalization between proteins and subcellular structures. Consistent with the reported finding, both the merged images and the line scanning results indicate significant colocalization between FrzCD and nucleoids (Figure 3G–I).

Using 2D analysis, scatterplots show significant intensity correlations between AglZ-GFP and AgmU-mCherry and between FrzCD-GFP and nucleoids (Figure 4B, H). These correlations are further confirmed by the high values of PCC, M1, and M2 (Figure 4C, I). In contrast, the scatterplot obtained from FrzCD-GFP and AgmU-mCherry does not show significant correlation (Figure 4E), which is consistent with the low values of PCC (0.43), M1 (0), and M2 (0) (Figure 4F). In contrast, the mean values of M1 (FrzCD, 0.6), M2 (nucleoid, 0.5), and PCC (0.75) from 42 cells in 20 images confirm significant overlap between FrzCD-GFP and nucleoid on the population level (Figure 5). Using coAnalyzer, we have validated the conclusion regarding colocalization in several publications [11–13].

Lbx1 is a transcription factor marker of dI4-dI6 neurons. In post-mitotic neurons at 10 days in vitro, neurons expressing Lbx1 only account for approximately 15% of DAPI-stained neurons [14]. Figures 3J and 4J show the localization between Lbx1-mCherry and DAPI-stained neurons in a mouse brain tissue [14]. While the scatterplot from individual ROIs shows a significant intensity correlation (Figure 3L), such a correlation does not exist at the population level (Figure 4K), which is confirmed by the low value of PCC (0.37) (Figure 4L). However, the calculated M1 (Lbx1) is 0.80, indicating that most of Lbx1-mCherry expressed neurons overlap with DAPI-stained neurons. In contrast, the calculated M2 (DAPI) is 0.14 (Figure 4L), indicating that only a small population (14%) of the DAPI-stained neurons express Lbx1-mCherry. Taken together, coAnalyzer generates reliable scatterplots and colocalization coefficients in both single-cell and multicellular samples. Importantly, besides colocalization analysis, coAnalyzer could be used to determine the expression patterns of proteins in multicellular samples.

To demonstrate the ability of coAnalyzer on highly complex localization patterns, we analyzed the colocalization between PDHA1 and mitochondria in *Caenorhabditis elegans*. PDHA1 is a subunit of pyruvate dehydrogenase E1, which is a component of the pyruvate dehydrogenase complex that localizes in the mitochondrial matrix. To validate the ability of coAnalyzer in analyzing signals in complex, irregular shapes, we obtained confocal microscopy images where PDHA1 and mitochondria were labeled with YFP and CFP, respectively [15]. Due to the cylindrical shape of *C. elegans*, both PDHA1 and mitochondria were illuminated unevenly (Figure 6A). It is thus difficult to analyze colocalization using the entire illuminated regions. To separate the fluorescence signals from their local backgrounds, we selected 16 small ROIs (Figure 6A). coAnalyzer can calculate M1, M2, and PCC for each ROI and combine all the data together (Figure 6B). The mean values of M1 (mitochondria) and M2 (PDHA1) are 0.60 and 0.66, respectively (Figure 6B), indicating that there is significant overlap between PDHA1 and mitochondria. However, the mean value of PCC is 0.53, indicating that PDHA1 does not occupy the entire mitochondria. Taken together, our results indicate that through the selection of multiple ROIs, coAnalyzer can quantify colocalization on complex images.

The results described above were generally consistent with those obtained using other colocalization analysis tools, such as Coloc2 (https://imagej.net/plugins/coloc-2) and JACoP (https://imagej.net/plugins/jacop). However, colocalization coefficients may differ across software packages due to variations in ROI selection, thresholding methods, and other analytical parameters. Therefore, to ensure reliable statistical comparisons among samples, we strongly recommend performing all analyses using the same software and processing workflow.

**Figure 6.**
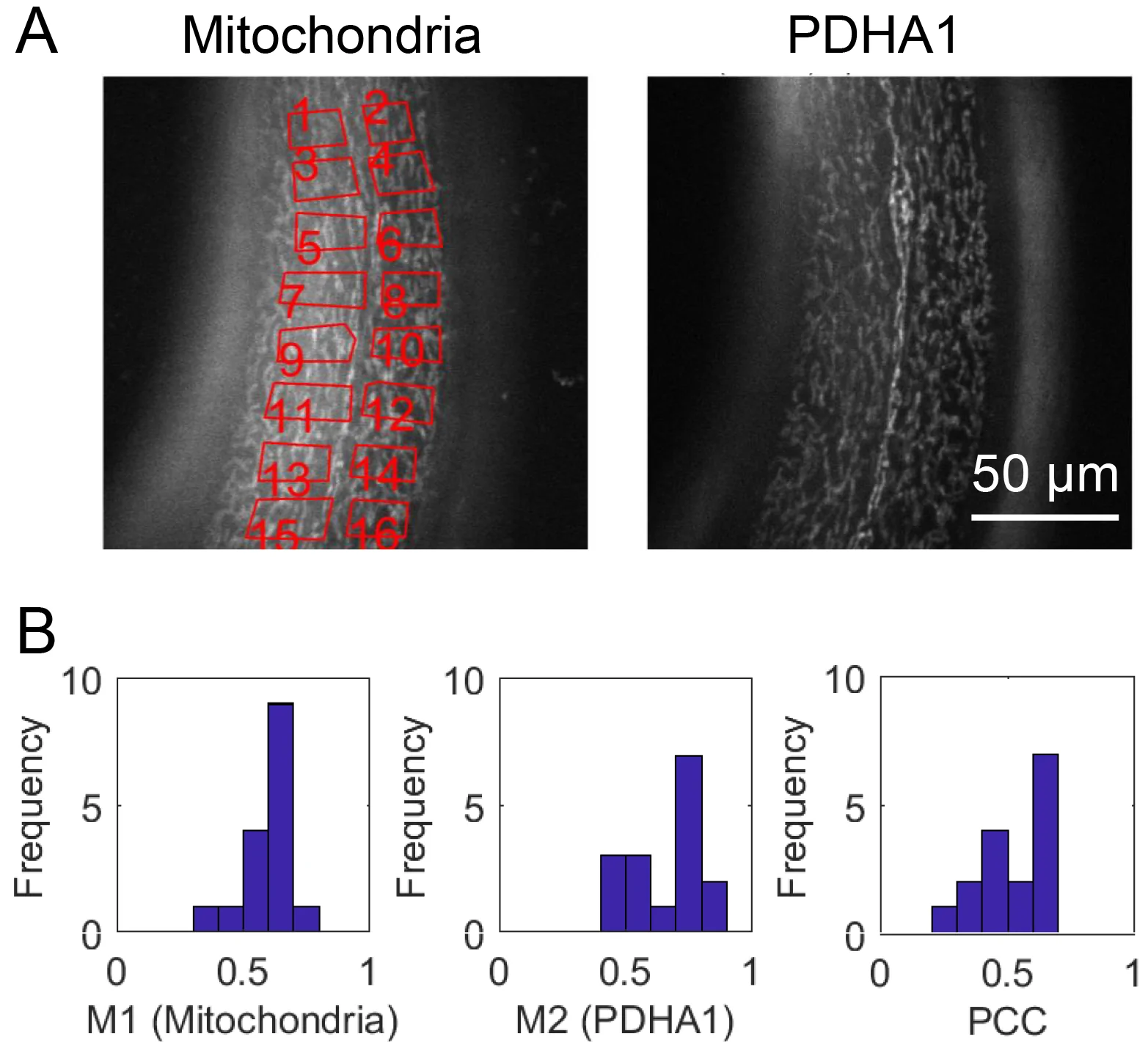
Colocalization quantification of PDHA1 and mitochondria by multi-ROI selection. (A) Fluorescent images of PDHA1-YFP and CFP labeled mitochondria in live *C. elegans* [15]. PDHA1 localizes in the mitochondrial matrix, where it serves as the catalytic E1 alpha subunit of the pyruvate dehydrogenase complex. Sixteen manually selected ROIs are outlined in red color. (B) Histograms of M1, M2, and PCC from the 16 ROIs.

## General notes and troubleshooting

**General notes**

1. Due to the diffraction of light, the resolution of fluorescence microscopy is limited to 200–250 nm [16], much larger than the distance between interacting macromolecules [17]. For this reason, overlapping fluorescence signals from different macromolecules do not guarantee interactions. Thus, conventional fluorescence microscopy usually serves as an auxiliary or screening tool in the studies of molecular interactions. Interactions at the molecular level must be confirmed by genetic, biochemical, biophysical, and super-resolution microscopy methods [18].

2. Although coAnalyzer provides an all-in-one package for image analysis, it is critical to select the right methods for the presentation of colocalization. For qualitative visualization, the image merging and line scanning functions will be sufficient. If proteins and/or structures show irregular patterns and no single line reflects their distributions, researchers could select ROIs and use the scatterplots from ROIs to present intensity correlations.

3. In contrast, M1, M2, and PCC provide quantitative information for colocalization. These coefficients become the only choices when researchers combine data from multiple ROIs and images. Compared with M1 and M2, which directly measure the spatial overlap, PCC also quantifies the correlation between the intensities of two fluorophores. While high PCC values confirm strong colocalization, low PCC values do not suggest the lack of colocalization. As shown in Figures 4 and 6, partial colocalization typically results in low PCC values. In such cases, M1 and M2 become better choices in the presentation and quantification of colocalization.

**Troubleshooting**

**Problem 1:** The GUI of coAnalyzer fails to launch.

Possible cause: The coAnalyzer folder is not in the MATLAB path.

Solution: Add the folder path of coAnalyzer to the MATLAB path and run the “co3.fig” figure file.

**Problem 2:** Unable to select ROI(s) in the ROI selection window.

Possible cause: The Image Processing Toolbox and the Optimization Toolbox are not installed.

Solution: Install the Image Processing Toolbox and the Optimization Toolbox in MATLAB before running the program.

**Problem 3:** The program displays the error message *“error, image ‘x’ does not exist”* when the user clicks the *Select ROI* button.

Possible cause: There is a mistake in the folder paths for images from one or both imaging channels.

Solution: Type the right folder paths for the *Color 1 folder* and the *Color 2 folder*.

**Problem 4:** There is a mismatch in the image files across image channels in the ROI selection window (Figure 2).

Possible cause: There is an error in the order of images in the image folders.

Solution: Correct the order of images in the image folders by renaming image files correctly in the right order of choice.

**Problem 5:** The program displays the error message *“error, data for image ‘x’ does/do not exist”* or displays partial results when the user clicks the *Combine* button.

Possible cause: The user has typed a wrong image range, or the user has not analyzed specific image/ROI numbers within the image range before clicking the *Combine* button.

Solution: The user should type the correct image range, or the user should analyze all image/ROI numbers within the image range to display the full combined results.
